# A novel videoscope and tool kit for percutaneous pericardial access under direct visualization

**DOI:** 10.1186/s12938-023-01085-z

**Published:** 2023-02-28

**Authors:** Justin D. Opfermann, Jacqueline M. Contento, Paige N. Mass, Axel Krieger, Charles I. Berul, Rohan N. Kumthekar

**Affiliations:** 1 Department of Mechanical Engineering, Johns Hopkins University, 3400 N. Charles Street, Baltimore, MD 21218 USA; 2 Sheikh Zayed Institute for Pediatric Surgical Innovation, Children’s National Hospital, Washington, USA; 3 Division of Cardiology, Children’s National Hospital, Washington, USA; 4 George Washington School of Medicine, Washington, USA; 5 Division of Cardiology, Nationwide Children’s Hospital, Columbus, USA; 6 Department of Pediatrics, The Ohio State University College of Medicine, Columbus, USA

**Keywords:** Pericardial access, Direct visualization, Micro-CMOS, Biomedical engineering, Percutaneous, Videoscope

## Abstract

**Background:**

Pericardial access is necessary for the application of epicardial cardiac therapies including ablation catheters, pacing and defibrillation leads, and left atrial appendage closure systems. Pericardial access under fluoroscopic guidance is difficult in patients without pericardial effusions and may result in coronary artery damage, ventricular injury, or perforation with potentially life-threatening pericardial bleeding in up to 10% of cases. There is a clinical need for a pericardial access technique to safely deliver epicardial cardiac therapies.

**Methods:**

In this paper, we describe the design and evaluation of a novel videoscope and tool kit to percutaneously access the pericardial space under direct visualization. Imaging is performed by a micro-CMOS camera with an automatic gain adjustment software to prevent image saturation. Imaging quality is quantified using known optical targets, while tool performance is evaluated in pediatric insufflation and pericardial access simulators. Device safety and efficacy is demonstrated by infant porcine preclinical studies (*N* = 6).

**Results:**

The videoscope has a resolution of 400 × 400 pixels, imaging rate of 30 frames per second, and fits within the lumen of a 14G needle. The tool can resolve features smaller than 39.4 µm, achieves a magnification of 24x, and has a maximum of 3.5% distortion within the field of view. Successful pericardial access was achieved in pediatric simulators and acute in vivo animal studies. During in vivo testing, it took the electrophysiologist an average of 66.83 ± 32.86 s to insert the pericardial access tool into the thoracic space and visualize the heart. After visualizing the heart, it took an average of 136.67 ± 80.63 s to access the pericardial space under direct visualization. The total time to pericardial access measured from needle insertion was 6.7 × quicker than pericardial access using alternative direct visualization techniques. There was no incidence of ventricular perforation.

**Conclusions:**

Percutaneous pericardial access under direct visualization is a promising technique to access the pericardial space without complications in simulated and in vivo animal models.

**Supplementary Information:**

The online version contains supplementary material available at 10.1186/s12938-023-01085-z.

## Background

The pericardium is a double-layered tissue that plays an important role in the protection and functionality of the heart [1]. The fibrous pericardium (outer layer) is composed of acellular and fibrous tissues that act as a physical barrier to isolate and prevent the spread of infection to the heart, while the serous pericardium (inner layer) lies directly on the epicardium and covers the great vessels [2]. The virtual space between the fibrous and serous pericardium is called the pericardial space and is filled with fluid to lubricate adjacent tissue layers when the heart is beating [3]. Because the pericardial space surrounds the heart, safely accessing this space is essential to deliver cardiac therapies and treatments to the epicardial surface of the heart as well as for pericardiocentesis to drain effusions and resolve cardiac tamponade [4].

Historically, non-surgical pericardial access (PA) was possible only when patients presented with cardiac and pleural effusions so that the fluid-filled pericardial space was easily observed via echocardiography [5] For patients with arrhythmias such as ventricular tachycardia (VT), PA was performed with open technique [6]. Sosa and colleagues were the first to perform non-surgical PA in patients with arrhythmias for epicardial mapping [7]. Using a subxiphoid approach and a spinal needle to introduce sterile fluid into the virtual space, they refined their technique to perform subxiphoid epicardial ablation under fluoroscopic guidance in patients with ventricular tachycardia [8]. Non-surgical PA has also been used for epicardial left atrial appendage closure systems [9] and as an alternative for delivering pacing leads for epicardial resynchronization therapy [10]. Today, non-surgical PA is essential for the practicing interventional electrophysiologist as the technique is used in up to 17% of adult VT ablation procedures at some centers, with cases expected to grow as the technique is more widely available [11].

Despite the frequency of non-surgical PA, the subxiphoid technique remains challenging as the avascular pericardial space is difficult to delineate using fluoroscopy without the presence of pericardial effusions. This limitation of fluoroscopic imaging can result in ventricular perforation and significant bleeding in up to 10% of cases [12, 13], which can be life threatening. In low-volume centers, complication rates rise an additional 26% as electrophysiologists are less practiced with the technique [14], and urgent on-site access to cardiothoracic surgery is highly recommended for any center when obtaining pericardial access. Despite these risks, the subxiphoid percutaneous approach remains the most widely used technique because it is the only strategy to offer unrestricted access to the pericardium [15].

Several technologies have been developed to reduce complication rates of non-surgical access. Notably, Kumar et al. developed a needle-in-needle technique to perform non-surgical PA under fluoroscopic guidance using a 21G needle so that accidental ventricular puncture would result in less bleeding [16]. However, this new technique did not reduce pericardial blood loss and users reported less tactile sensation during PA. In US Patent Application US20080294174A1, Bardsley et al. introduce a method and apparatus for pericardial access using a tube with elongating structures that pierce the parietal pericardium when it is in contact with the surface of the heart [17]. By retracting the tube from the heart, the parietal pericardium is separated from the visceral pericardium so that a needle can access the pericardial space. However, the tool is not intuitive to use, relies on fluoroscopy that exposes both patients and staff to ionizing radiation, and there are no reports that the tool has been used clinically. Alternatively, novel access tools that do not rely on fluoroscopy, such as the EpiAccess system, have been used in clinical trials [18]. This technology incorporates a pressure sensing element within the PA needle to provide real time feedback when the needle has entered the pericardial space. Similarly, Ludwig et al. describe a PA needle that used radiofrequency energy to access the pericardium under computerized axial tomography (CT) guidance in preclinical studies [19]. While both solutions effectively reduce the incidence of pericardial bleeding, they still do not address the limitations of image guidance during PA as the pericardial space and access needle cannot be imaged for the entire procedure.

To access the pericardial space more safely, our lab developed a multi-lumen tool and corresponding technique to obtain PA using a subxiphoid approach under direct visualization [20]. The tool has been used with a deflectable endoscope to implant cardioverter defibrillation leads [21], pacemaker leads [22], and prototype leadless pacemakers [23] within the pericardial space in acute and chronic animal studies. Additionally, pericardial adhesions do not preclude the direct visualization approach [24], as no differences were observed in the chronic performance of leads when compared to an open surgical technique [25]. While the combination of direct visualization with subxiphoid technique has been used successfully, the procedure requires a small 1-cm incision and the simultaneous coordination of two working channels to maintain visualization of access tools in the surgical field. In this paper, we address these limitations by presenting the design and evaluation of a novel percutaneous PA tool kit that eliminates the need for a multi-lumen tool and minimizes the entry size to a Veress needle. Direct visualization is achieved using a micro-Complementary Metal-Oxide Semiconductor (micro-CMOS) camera and ring illumination within a PA needle, so that the heart, great vessels, and pericardium are appropriately visualized by a single operator. The scientific contributions of this work include presentation of the tool design, quantification of the imaging performance in bench studies, and validation of the tool and technique to obtain pericardial access in a pediatric simulator, followed by an infant animal model. Using this access kit, non-surgical PA can be performed under direct visualization through only a needle puncture that may be especially beneficial for infant, neonate, and fetal epicardial or pericardial interventions.

## Experiments and results

### Camera and insufflation characterization

The videoscope’s optical performance was characterized by the following metrics: image resolution, geometric image distortion, image magnification, and automatic gain adjustment. Further evaluation of insufflation capability was conducted to demonstrate compatibility with thoracic surgery workflows.

### Image resolution

**Fig. 1:**
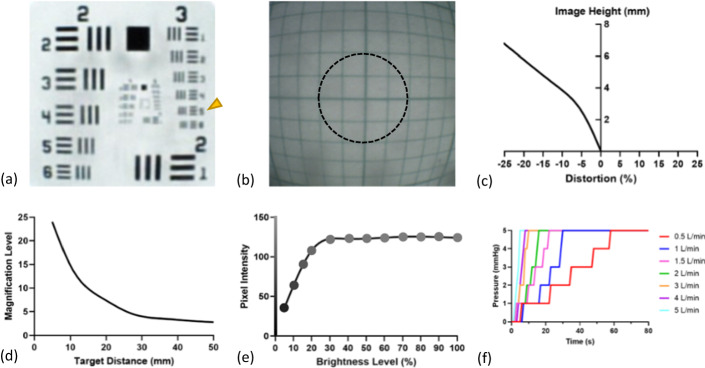
Imaging and insufflation results. Representative image of the USAF resolution target with smallest resolved pattern identified with an arrow (**a**), distortion target with 2-mm region of unnoticeable distortion identified by the dashed circle (**b**), the respective distortion curve as a percentage of image height (**c**), magnification performance as a function of distance (**d**), image intensity with representative grayscale values at different illumination levels (**e**), and insufflation performance for various flow rates (**f**)

Resolution was quantified using the 1951 USAF resolution target [26]. The target is split into six groups of six-line pairs that are progressively smaller. Each line pair is called an element of the target. The micro-CMOS camera was used to image the USAF target (Thorlabs, NJ) at 5 mm, which is the ideal working distance for the camera (Fig. 1a). Using Eq. (1), the camera resolution in $lp/mm$ (line pairs per millimeter) was calculated where *group* and *element* correspond to the smallest resolved line pair. As observed by the arrow in Fig. 1a, the maximum resolution was found to be 12.7lp/mm, which corresponds to a minimum detectable feature size of 39.37 μm:

1 $$Resolution \left(LP/mm\right)={2}^{group+\left(element-1\right)/6.}$$

### Geometric image distortion

Local geometric distortion was quantified in accordance with the International Organization for Standardization (ISO) document ISO 17850:2015 [27]. A distortion target made from a 20 mm x 20 mm sheet of 1 mm grid paper (Sakaeshigyo, Japan) was centered to the micro-CMOS camera and imaged at 5 mm. Using ImageJ (NIH, MD), the actual distance (*AD*) in *pixels* from the origin to each pair of intersecting grid lines was calculated. Similarly, the theoretical distance (*TD*) from each pair of intersection grid lines to the origin was calculated using the known 1-mm spacing of the distortion grid. Distortion as a percentage of image height was then calculated using *AD* and *TD* as in Eq. (2). Distortion percentages at the same theoretical distance were averaged together. A representative image of the distortion target is illustrated in Fig. 1b, and a graph of distortion percentage relative to image height is illustrated in Fig. 1c. It was noted that the image exhibited barrel distortion that was detectable along the edge, but not noticeable within the central 2 mm of the image (the area within the dashed circle):

2 $$Distortion \left(\mathrm{\%}\right)=\frac{AD-TD}{TD}\times 100\mathrm{\%}.$$

### Image magnification

To quantify the digital magnification across the tool’s depth of field, the distortion target was imaged at various intervals from 5–50 mm. At each interval, ImageJ was used to compute the image length (*IL*) in millimeters of the four nearest grid lines to the origin. Simultaneously, the camera’s image was displayed on a 27″ HD monitor and the screen length (*SL*) between the origin and four nearest grid lines was measured in millimeters using a set of calipers. Corresponding *IL* and *SL* measures were averaged together, and Eq. (3) was used to calculate the magnification at each interval. The magnification level is plotted against the target distance as illustrated in Fig. 1d and was found to be a maximum of 23.98 × at the ideal 5 mm working distance:

3 $$Magnification=\frac{SL}{IL}.$$

### Automatic gain adjustment

The micro-CMOS controller is equipped with an automatic gain adjustment to maintain a constant image brightness independent of lighting conditions. The setting works by automatically adjusting the image gain to limit the brightest pixel intensity once a threshold has been reached. This setting is especially helpful during in vivo applications where illumination and tissue glare can saturate the image. To experimentally verify this feature, a 5 cm x 5 cm white target was centered 5 mm from the end of the camera. The power of the illumination sheath was then adjusted in intervals from 5–100% with images taken at each interval. ImageJ was used to convert the images to grayscale and calculate the average intensity of pixels within the central 5 mm of the target. A PM130D power meter with S120C sensor (Thorlabs, NJ) was used to measure the illumination intensity throughout the test. Average pixel intensity for each power setting is plotted in Fig. 1e. Representative images of the grayscale target are shown at sample intensities.

### Insufflation performance

To evaluate insufflation performance, a commercial insufflator with carbon dioxide (STORZ, Germany) was connected to the insufflation Luer on the illumination sheath. The PA tool was inserted into the thoracic insufflation simulator and carbon dioxide (CO_2_) was used to inflate the plastic chamber. The maximum insufflation flow rate was adjusted from 0.5L/min to 5L/min and the time to reach a pressure of 5 mmHg was recorded. A pressure threshold of 5 mmHg was chosen as that is the thoracic pressure at which a patient’s hemodynamic response begins to worsen [28]. Insufflation pressure curves for each flow rate were recorded and plotted as shown in Fig. 1f.

### Pediatric pericardial access simulation

**Fig. 2:**
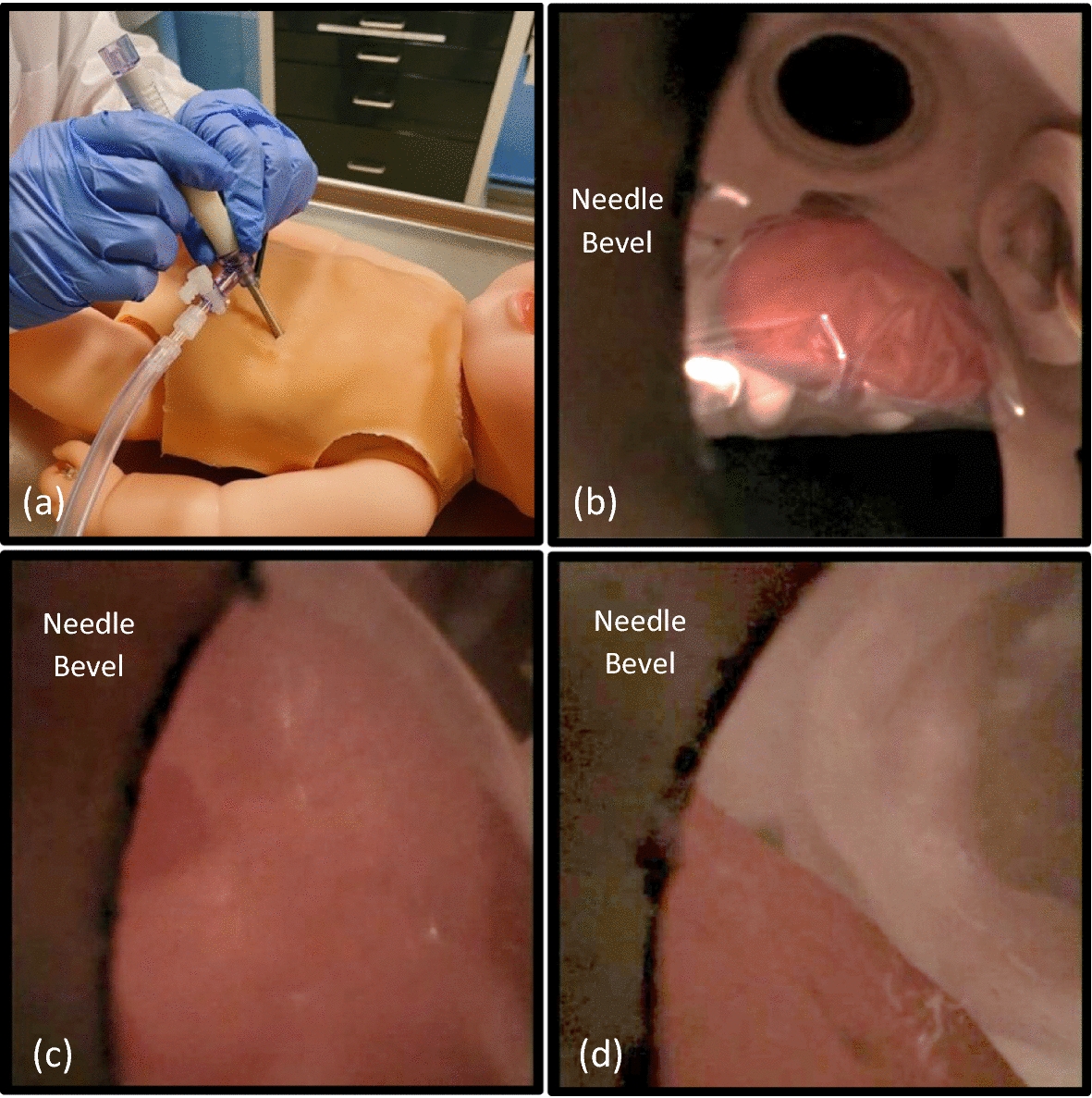
Pericardial access using the infant simulator. Inserting the pericardial access tool (**a**), visualizing the heart (**b**), tenting and separation of the pericardium from the epicardium marked by an arrow (**c**), viewing from inside the pericardial space (**d**)

To validate the procedure workflow, an expert user was asked to visualize and then access the pericardial space of the simulator. The PA tool was prepared by placing a Veress needle inside the illumination sheath and setting the illumination power to 100%. The user accessed the thoracic space by inserting the Veress needle and illumination sheath below and lateral to the xiphoid process (Fig. 2a). After removing the Veress needle, visualization of the heart was confirmed by inserting the 14G PA needle with embedded micro-CMOS camera through the illumination sheath (Fig. 2b). The distal end of the tool was rotated so that the needle’s bevel was on the left side of the image and the needle was advanced towards the apex of the heart. Pericardial access was achieved by tenting the synthetic pericardium using the needle’s bevel, and then advancing the needle under direct visualization (Fig. 2c). Pericardial access was confirmed when the synthetic pericardium no longer blocked the camera’s view of the epicardium (Fig. 2d). After accessing the pericardial space, the user manipulated the access needle with the camera to visualize the pericardial space. The time from visualization to pericardial access was 38 s.

### In vivo feasibility testing

**Fig. 3:**
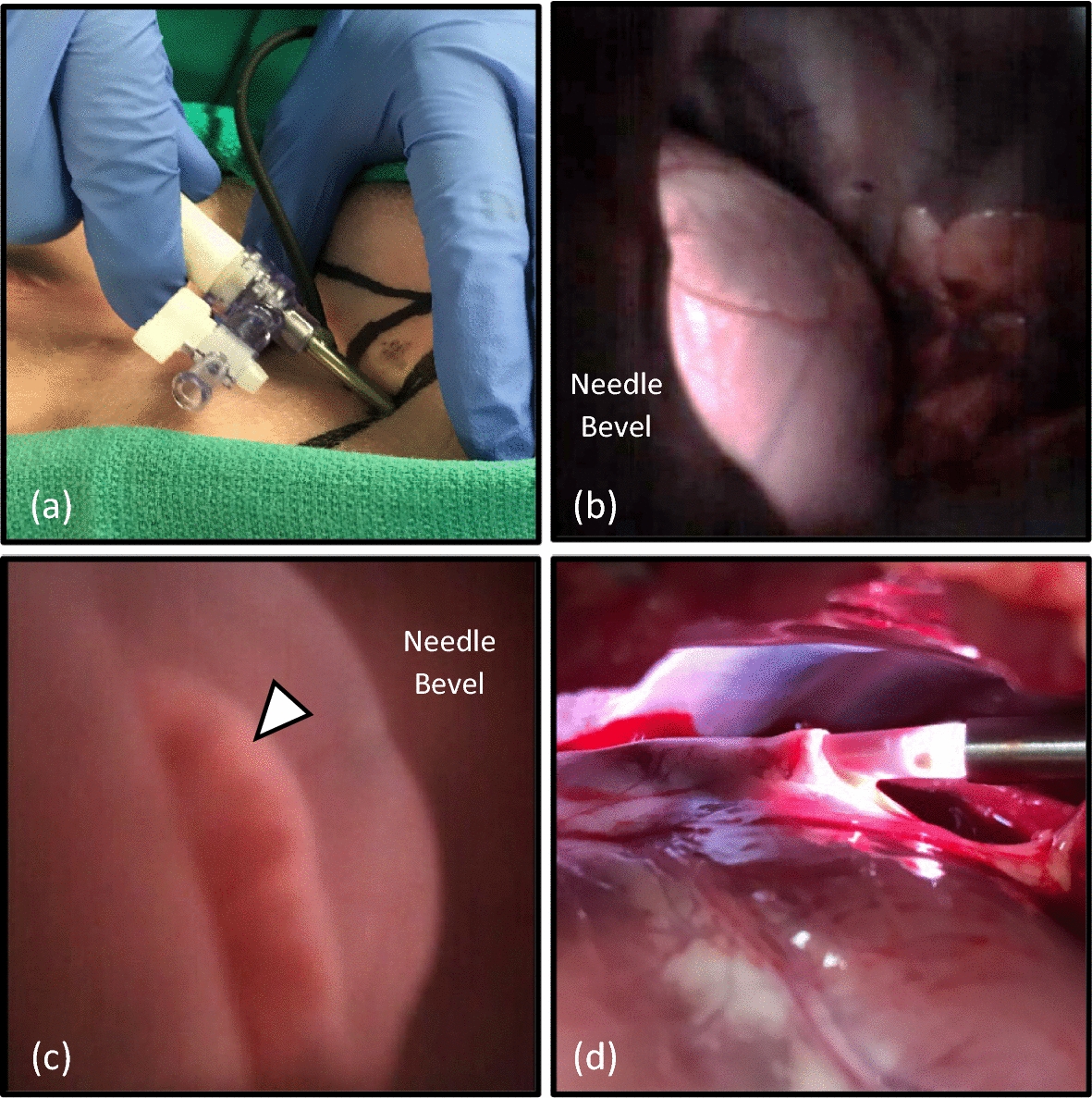
Preclinical pericardial access. Inserting the pericardial access tool (**a**), visualizing the heart and coronary arteries (**b**), viewing the left atrial appendage from inside the pericardial space (arrow) (**c**), introducer in the pericardial space (**d**)

An infant porcine model (*N* = 6) was selected for in vivo studies due to similarity of coronary artery anatomy and relative size to the human heart [29]. For each procedure, a pediatric cardiac electrophysiologist made a 2-mm nick in the skin of the animal and inserted the illumination sheath with a Veress needle into the thoracic space. Insufflation with carbon dioxide was administered at a flow rate of 2.5L/min until a pressure of 4 mmHg was achieved. The PA needle with embedded micro-CMOS camera was inserted through the illumination sheath and used to visualize the heart with associated blood vessels (Fig. 3a). The illumination power was adjusted to minimize glare from the surface of the pericardial tissue, and the bevel of the needle was oriented to the left of the screen. Following the same technique practiced in the pediatric simulator, the pericardial space was accessed under direct visualization (Fig. 3b). Manipulation of the needle enabled imaging of the pericardial space including the left atrial appendage (Fig. 3c). Following pericardial access, a guidewire was passed through the needle to maintain access pericardial space so the needle could be removed. After the needle was removed, an 8Fr introducer was loaded onto the guidewire and advanced through the illumination sheath into the pericardial space. Placement of the introducer was confirmed during necropsy (Fig. 3d). It took the electrophysiologist an average of 66.83 ± 32.86 s to insert the PA tool into the thoracic space and visualize the heart, and then 136.67 ± 80.63 s to access the pericardial space under direct visualization. There were no incidents of ventricular perforation.

## Discussion

The novel tool kit presented in this paper offers an alternative approach to non-surgical PA. Using a micro-CMOS camera, the tool achieves direct visualization, insufflation, and illumination performance that exceeds clinical requirements. Image quality is standardized by imaging known optical targets and following ISO specifications. The system can detect features as small as 39.37 μm and is capable of 23.98 × digital magnification. These results indicate key epicardial structures such as the coronary arteries and associate branch vessels can be resolved as was observed during the in vivo study [30]. Of note, barrel distortion was present in the image, which is common in micro-optical applications and typically un-noticed if less than 3% [31]. Distortion of the needle was calculated to be 3.5% and did not preclude pericardial access, however image processing can remove distortion across the entire image in real time for future applications [32]. Insufflation is also sufficient for clinical use as the tool sustains 5 mmHg of pressure for flow rates from 0.5L/–5L/min.

**Fig. 4:**
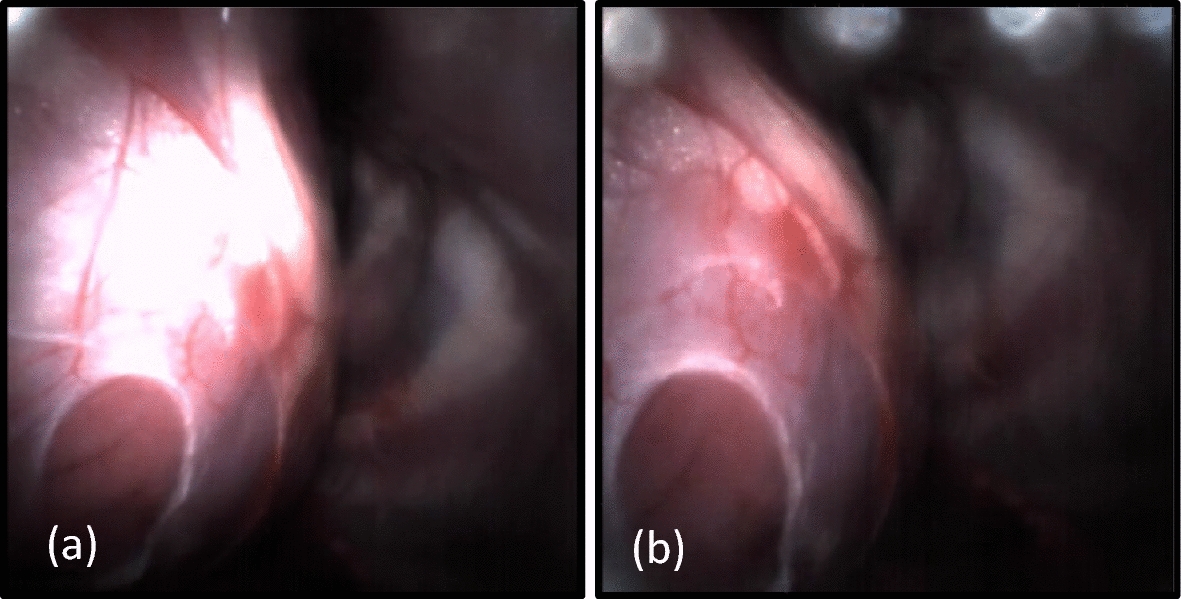
View of the heart, epicardial structures, and associated glare without a linear polarizer (**a**) and with application of a linear polarizer (**b**)

During the in vivo study, the tool’s automatic gain adjustment was found to be particularly useful as reflections from pericardial tissue were enough to saturate the image at the lowest illumination. After applying automatic gain adjustment to the system, the image brightness was scaled in real time so that the heart and needle were constantly visible. While the presence of reflections did not prohibit pericardial access, the resulting glare was noticeable if the illumination sheath was too close to the pericardium (Fig. 4a). During necropsy of the final animal procedure, we attempted to remove the glare in situ by using a pair of 1 cm x 1 cm linear polarizing sheets affixed to the distal ends of the illumination sheath and micro-CMOS camera. While the polarizing sheets were too large to be used with the percutaneous system in vivo, the polarizers were effective at reducing the perceived glare as shown in Fig. 4b. A micro-polarizing beam splitter will be integrated with the access tool in future work so that the effects of polarizers on glare reduction can be studied in vivo. Glare was not noticeable in the pediatric phantoms that use synthetic tissues with fewer reflections.

Furthermore, a percutaneous technique for pericardial access has several advantages as compared to our previous subxiphoid approach [20]. First, the percutaneous approach enables miniaturization of the technique from a 12-mm skin incision to a 4-mm skin incision. The shorter incision length is desirable as it can be closed without the need for sutures, and results in less inflammatory and immune response in the patient [33]. Second, our prior tool and technique used a fixed approach angle to implant epicardial pacing and defibrillation leads in a limited epicardial region. A percutaneous approach, however, enables pericardial access from multiple trajectories which expands utility of the technique to epicardial ablation; a procedure where a catheter must be directed to multiple epicardial sites. Finally, the percutaneous approach employs an inline visualization strategy to improve control and visualization. In [20], the camera and access needle are crossed with respect to each other which requires the user to learn how to mirror hand movements to keep the needle in view of the camera. The percutaneous tool solves this problem by coupling the camera to the access needle so that the bevel always remains centered in the camera view and visualization control is maintained with an intuitive motion.

The impact of improved usability due to percutaneous inline visualization is highlighted by direct comparison with our alternative subxiphoid approach. Using direct visualization and two access ports through a single incision, Clark demonstrated it was possible to visualize the heart and access the pericardial space in 22.75 min [21]. Notably, nearly 95% of the time needed for pericardial access using this approach was spent to insert the single port tool and manipulate two access ports for correct visualization of the heart. In this paper, we demonstrated that a percutaneous approach simplifies the procedure such that visualization of the heart can be achieved in just over one minute, with pericardial access occurring just 3.39 min into the procedure. This timing is 6.7 × quicker than pericardial access using alternative direct visualization techniques [21–25]. Furthermore, by demonstrating capability of the videoscope to insert an 8Fr introducer into the pericardial space, it would be trivial to expand the functionality of the videoscope to include delivery of pacing leads and ablation catheters to the epicardial surface of the heart in future studies.

Safe pericardial access is an essential skill for the practicing electrophysiologist. A subxiphoid percutaneous technique is required for patients that need epicardial ablation or implantation of left atrial appendage closure systems. As users become more familiar with the technique, the eligible patient population will continue to expand. Elderly patients with tortuous vasculature, adults with insufficient coronary sinus branch vessels on the left ventricle, and an estimated 10,000 pediatric patients each year could benefit from a percutaneous approach to implant pacing therapies [34]. Clinical trends motivate the need for novel tools and techniques for pericardial access as ventricular perforation and pericardial bleeding remain a significant concern during currently utilized fluoroscopic guidance, while damaging effects of fluoroscopic radiation are particularly concerning in vulnerable populations [35]. While this paper is limited to bench evaluation and preclinical testing, the percutaneous videoscope is a first step towards safe pericardial access. Future studies to implant pacing leads and ablation catheters in the pericardial space are necessary prior to human trials. By addressing challenges associated with visualization, illumination, and insufflation, direct visualization may be a viable alternative to fluoroscopy for percutaneous pericardial access.

## Conclusions

In this paper, we reported the design and evaluation of a novel percutaneous pericardial access tool. Using micro-CMOS technology, the tool achieved high-quality imaging to visualize the heart, coronary arteries, and pericardial space while precisely entering the pericardial space. The tool’s performance was evaluated in bench testing, and the procedure workflow was validated by an expert user in a pediatric phantom. An electrophysiologist demonstrated clinical feasibility by accessing the pericardial space in an in vivo model. As imaging resolution improves in smaller physical footprints, direct visualization with micro-CMOS cameras will play an important role in the delivery of epicardial therapies.

## Methods

### Design of the pericardial access tool

**Fig. 5:**
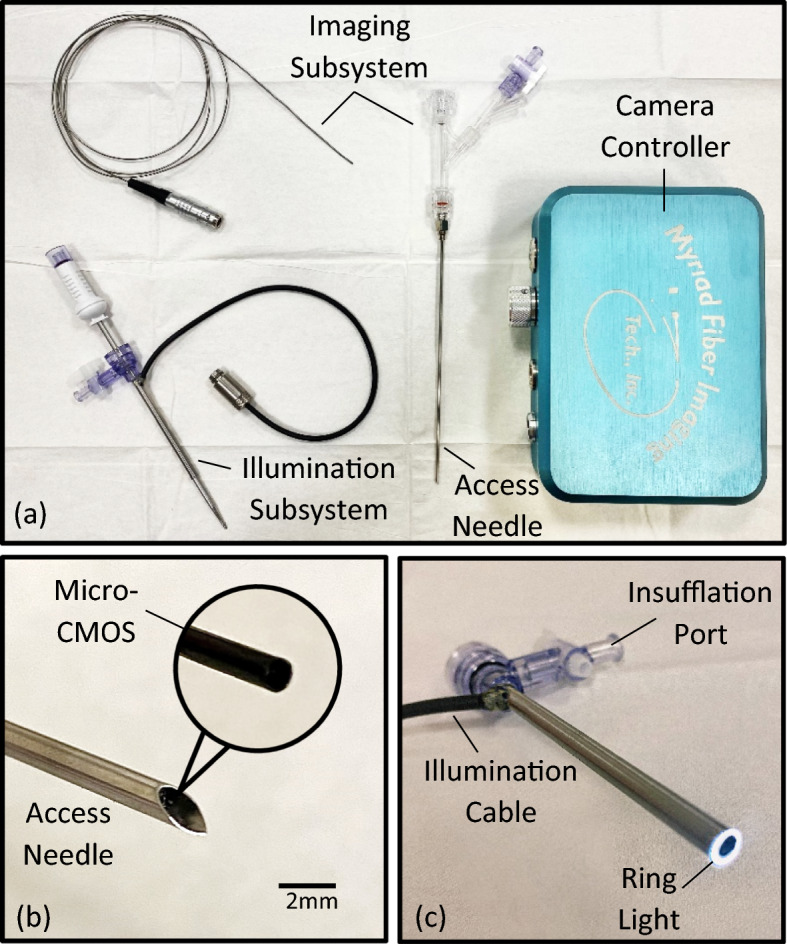
The novel pericardial access tool with key subsystems (**a**), including access needle with micro-CMOS camera (**b**), and illumination sheath (**c**)

Intraoperative imaging is performed using a series of relay lenses (boroscope) or fiber optics (fiberscope) to transmit light from inside the body to the user’s eye. For high-quality imaging, boroscopes are preferred since fiberscopes suffer from pixilation and optical cross talk that can reduce image contrast, resolution, and induce a honeycomb pattern in the image [36]. However, for small diameter and flexible applications, fiberscopes are traditionally preferred as machining micro-optical relay lenses is expensive, and small boroscope apertures limit illumination efficiency resulting in darker images [37]. Since accessing the pericardial space requires high-resolution imaging within small diameters, neither boroscopes nor fiberscopes are ideal for the procedure. Thus, we choose to utilize micro-CMOS imaging technology to overcome these clinical requirements for direct visualization PA. The micro-CMOS imager is packaged with illumination and insufflation to create a novel videoscope tool kit for pericardial access. Figure 5a illustrates the PA tool with the visualization, illumination, and insufflation subsystems highlighted.

### Imaging subsystem

Imaging is achieved using a micro-CMOS sensor with 400 × 400 pixels (OVM6946, OmniVision, CA). A glass lens provides a 90° field of view (FOV), and the assembly is packaged within a 1050 × 1050 x 2266 μm housing (Fig. 5b). The maximum frame rate is 30 frames per second, which is fast enough to image moving structures such as a beating heart without a perceived delay [38]. The camera is surrounded by cladding for moisture protection and terminated with a coaxial cable. A custom controller and software (OasisCAM066, Myriad Fiber, MA) is used to convert the camera signal to USB output, vary the image gain, provide digital zoom, and record video images. Overall, the micro-CMOS scope is 1.6 mm in diameter, 1 m long, has a 5-mm working distance, and has a 3-50 mm depth of field.

A 14G needle is used for PA in this study (Vita Needle, MA). The needle is sharpened with a 30° lancet tip and has a standard luer hub. The camera is housed within the lumen of the needle, positioned proximal to the bevel, and oriented such that the image axis is normal to the surgical field (Fig. 5b). The camera’s position and orientation are fixed using a hemostasis valve with Y-connector (Qosina, NY) that is attached to the needle’s hub. The angle between the needle’s bevel and the camera can be manually adjusted by rotating the distal end of the valve.

### Illumination and insufflation subsystems

A custom illumination and insufflation sheath was manufactured for this study (Myriad Fiber, MA) (Fig. 5c). The sheath consists of an insufflation needle (Ethicon, NJ) that is surrounded by optical fiber and a stainless-steel tube. The optical fiber is terminated with an ACMI fitting and adapter so that the sheath can be coupled to a STORZ fiber guide. Illumination is provided by a STORZ light box that has adjustable output from 5–100%. As observed in Fig. 5c, the optical fibers are arranged as a ring light at the distal end of the sheath to optimize illumination of the surgical field. Insufflation can be administered through the sheath by connecting carbon dioxide to the Luer lock on the proximal hub. Insufflation pressures and flow rates can then be adjusted using a commercial insufflator. The illumination sheath is compatible with a standard Veress needle for thoracic insertion.

### Surgical workflow

**Fig. 6:**
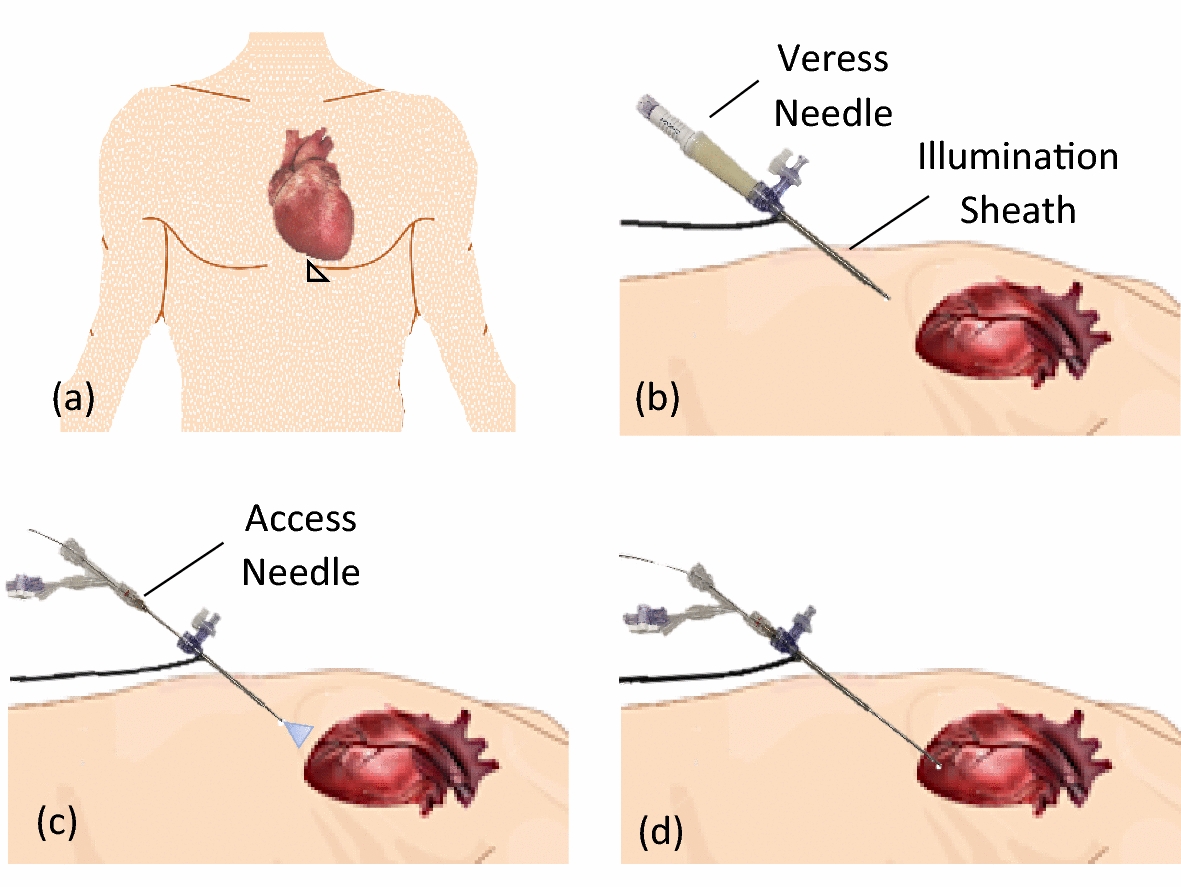
Surgical workflow to use the pericardial access tool, including identification of a subxiphoid and lateral region (**a**), insertion of the illumination sheath (**b**), visualization of the heart and epicardial structures (**c**), and access to the pericardial space (**d**)

Figure 6 illustrates the surgical workflow for gaining access to the pericardial space under direct visualization. First, the operator identifies a region subxiphoid and lateral as indicated by the triangular region in Fig. 6a. The user then inserts the Veress needle and illumination sheath assembly while aiming laterally towards the chest wall (Fig. 6b). After administering insufflation with CO_2_, the user removes the Veress needle and inserts the 14G access needle with embedded micro-CMOS camera to visualize the heart (Fig. 6c). The pericardial space is then accessed under direct visualization while care is taken to avoid key epicardial structures such as the coronary arteries (Fig. 6d). After pericardial access is achieved, a 0.035-in. guidewire can be passed through the needle and into the pericardial space. After inserting the guidewire, the needle is removed from the patient and an introducer used to deliver either a pacing lead or a catheter for epicardial ablation can be inserted into the pericardial space using an over-the-wire technique.

## Experimental testbeds

### Light-controlled camera box

A box built from 80/20 aluminum tubing and draped with blackout cloth was used to control lighting conditions, standardize testing protocols, and replicate the dark environment inside of a body. The PA tool was mounted within the box such that the camera plane was parallel to imaging targets. The vertical position of the tool could be adjusted as needed and illumination was set to 100% for all tests unless noted as in the Automatic Gain Threshold Experiments.

### Pericardial access and thoracic insufflation simulators

**Fig. 7:**
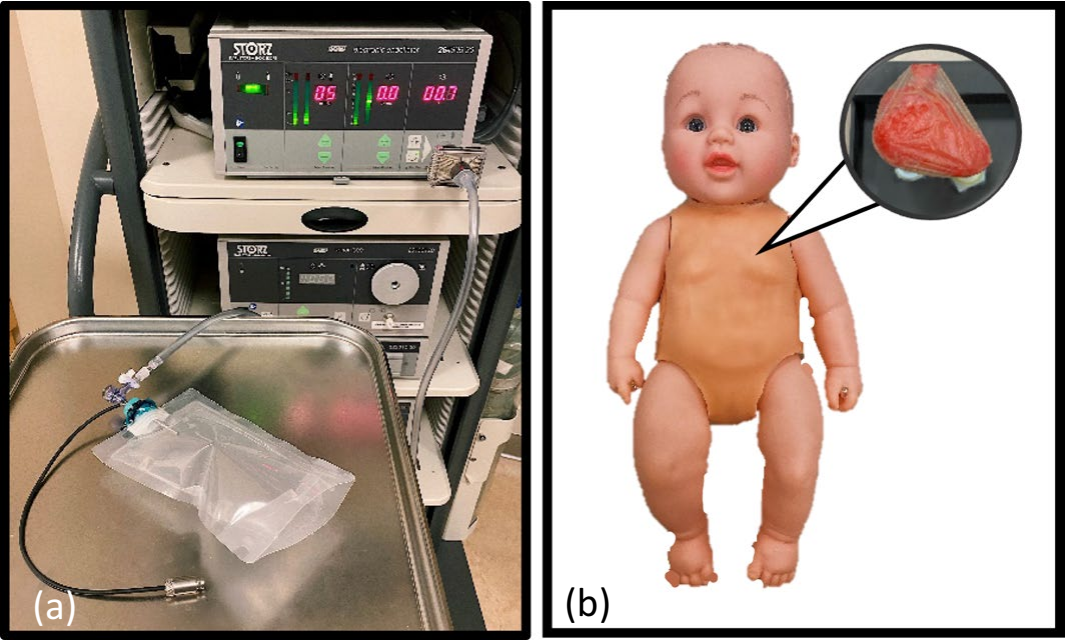
Images of the pediatric surgical simulators with the insufflation simulator connected to a commercial insufflator shown in (**a**) and the thoracic simulator with corresponding heart and pericardium shown in (**b**)

To characterize insufflation performance, a thoracic insufflation simulator was assembled by sealing a 500-mL chamber with a silicone gasket (Fig. 7a). The chamber was sized to mimic the thoracic volume of a 2-month-old patient [39, 40], and the chamber’s edges were heat welded to prevent air leakage during the experiments. The silicone gasket was taken from a commercial gel port (Applied Medical, CA) designed to maintain insufflation when pierced by surgical tools. A second simulator was fabricated to enable pediatric pericardial access (Fig. 7b) [41]. The heart was 3D printed using segmented clinical CT scans and surrounded by a clear plastic film representing the pericardium. The assembly was placed within the thorax of a plastic doll covered in simulated skin tissue (Smooth-on, PA).

### Preclinical animal model

An infant porcine model was selected due to similarity of coronary artery anatomy, relative size to the human heart, and to match previous pericardial access experiments [21–25]. The animals averaged 4.0 kg in weight, which corresponded to the thoracic volume of the pediatric simulators. The animals were sedated with intramuscular ketamine (20 mg/kg) and xylazine (2 mg/kg) and anesthetized with vaporized isoflurane (1–5%). The animals were positioned supine, and a mechanical ventilator was used to maintain breathing. The animals were draped, and the xiphoid process was marked with black pen. Each animal’s color, heart rate, respiration, and blood oxygen level were monitored. This experiment was approved by the institutional animal care and use committees at Nationwide Children’s Hospital (IACUC AR20-00155) and Children’s National Hospital (IACUC 30442).

## Supplementary Information

**Additional file 1:** Percutaneous pericardial access under direct visualization in a porcine model using the novel videoscope and access kit.

## Data Availability

The datasets used and/or analyzed during the current study are available from the corresponding author on reasonable request.

## Abbreviations

PA: Pericardial access
VT: Ventricular tachycardia
CT: Computerized axial tomography
Micro-CMOS: Micro-complementary metal-oxide semiconductor
CO_2_: Carbon dioxide
ISO: International Organization for Standardization
FOV: Field of view
